# HflX mediates rifampicin resistance in *Brucella* by downregulating the expression of RNA polymerase-associated genes

**DOI:** 10.3389/fmicb.2026.1823866

**Published:** 2026-04-15

**Authors:** Hao Geng, Mengzhu Qi, Mengru Su, Feijie Zhi, Yuefeng Chu

**Affiliations:** 1 State Key Laboratory for Animal Disease Control and Prevention, Lanzhou Veterinary Research Institute, Chinese Academy of Agricultural Sciences, College of Veterinary Medicine, Lanzhou University, Lanzhou, China; 2 Gansu Province Research Center for Basic Disciplines of Pathogen Biology, Lanzhou, China; 3 Key Laboratory of Veterinary Etiological Biology, Key Laboratory of Ruminant Disease Prevention and Control (West), Ministry of Agriculture and Rural Affairs, Lanzhou, China

**Keywords:** *Brucella*, HflX, rifampicin resistance, RNA polymerase, stress response

## Abstract

Brucella species (*Brucella* spp.) are facultative intracellular zoonotic pathogens responsible for brucellosis, a disease causing substantial global public health and economic burdens. Rifampicin remains a first-line therapeutic agent, but the molecular mechanisms underlying rifampicin resistance in *Brucella* remain poorly defined, especially the contribution of ribosome-associated regulatory proteins. HflX is a conserved ribosome-binding GTPase involved in ribosomal quality control and antibiotic resistance, yet its role in rifampicin resistance has not been reported. Here, we constructed *hflX* deletion and complemented strains of *Brucella* abortus 2308 and characterized their phenotypes using antimicrobial susceptibility tests, growth and time-kill assays, electron microscopy, proteomics, and RT-qPCR. Deletion of *hflX* significantly increased bacterial susceptibility to rifampicin, impaired growth recovery, and intensified intracellular stress without disrupting cell envelope integrity. Mechanistically, *hflX* depletion led to coordinated downregulation of RNA polymerase (RNAP) core subunits (*rpoA*, *rpoB*, and *rpoC*) and σ factors (*rpoD* and *rpoH*) at both protein and mRNA levels. Our findings demonstrate that HflX mediates rifampicin resistance in *Brucella* by regulating RNAP-associated gene expression and metabolic adaptation, establishing a novel HflX–RNAP regulatory axis. This study expands the understanding of antibiotic resistance in intracellular pathogens and highlights HflX as a promising target for developing anti-resistance strategies against brucellosis.

## 1 Introduction

Brucellosis is a widespread zoonotic disease caused by *Brucella* spp. Globally, brucellosis poses a significant public health and veterinary burden, with an estimated 1.6–2.1 million new human cases each year (Laine et al., 2023). The incidence of the disease exhibits a broad geographic distribution, with over 170 countries having reported confirmed human brucellosis cases (Liu S. et al., 2024). Regions with the highest prevalence include the Mediterranean Basin, the Middle East, Latin America, Asia, and parts of Africa (Qureshi et al., 2023). Unfortunately, the emergence and spread of rifampicin-resistant *Brucella* strains pose a severe threat to treatment efficacy, leading to prolonged illness, increased risk of relapse, and higher healthcare costs. Despite its clinical importance, the underlying molecular mechanisms of rifampicin resistance in *Brucella* remain poorly characterized.

Rifampicin serves as a first-line treatment for brucellosis. Its antibacterial properties are attributed to its high affinity for a conserved pocket in the β subunit (RpoB) of bacterial RNAP, where rifampicin binding sterically blocks extension of the nascent RNA beyond 2–3 nucleotides, thereby inhibiting initiation of transcription (Mosaei and Zenkin, 2020). Rifampicin resistance primarily arises from amino acid substitutions in the rifampicin-resistance-determining region (RRDR) of *rpoB*. These mutations reduce the drug’s affinity for RNAP by inducing subtle conformational changes in the drug-binding pocket (Marianelli et al., 2004; Molodtsov et al., 2017). In addition to mutations, *Brucella* exhibits an adaptive response to low-dose rifampicin during the early growth phase (Yang et al., 2020). In the absence of *rpoB* mutations, rifampicin resistance is associated with alterations in the VirB operon (with a central role for VirB7–11), ABC transporters, β-lactam resistance genes, quorum-sensing systems, and genes involved in DNA repair and replication (Ma et al., 2023; Valdezate et al., 2010). Additionally, the (p)ppGpp synthase Rsh has been shown to promote rifampicin resistance in *Brucella abortus* by positively regulating the type II toxin-antitoxin module (mbcTA) (Liu et al., 2023; Liu S. et al., 2024). However, all these reported resistance-related factors are unrelated to ribosomal quality control proteins, and the role of ribosome-associated regulatory factors in *Brucella* rifampicin resistance has not been explored to date, leaving a critical gap in the comprehensive understanding of *Brucella*’s antibiotic resistance regulatory network. These findings demonstrated that identifying novel, druggable targets for combating rifampicin resistance in *Brucella* can inform the surveillance and control of resistant strains. This need is driven by the mounting challenge of antibiotic resistance in the development of new antibacterial agents.

HflX is a broadly conserved, ribosome-associated P-loop GTPase, with ATPase activity reported in certain species. It functions as a key factor in ribosomal quality control under stress conditions, including heat shock and hypoxia (Dey et al., 2018). HflX recognizes and dismantles drug- or stress-stalled 70S ribosomes, facilitating subunit recycling and thereby preserving translational homeostasis (Coatham et al., 2016; Rudra et al., 2020). Sequence alignment revealed that *Brucella* HflX also harbors a conserved N-terminal extension (NTE) homologous to that of *mycobacteria*, implying that this structural domain may contribute to the regulatory function of HflX in ribosome homeostasis, antibiotic susceptibility and intracellular adaptation characterized in this study (Majumdar et al., 2025; Seely et al., 2024). Conversely, *hflX* knock compromises cellular energetics and the translational state, thereby reducing the bacterial resistance to various antibiotics (Koller et al., 2022). Notably, all reported antibiotic resistance mediated by HflX is targeted at protein synthesis inhibitors, and there is no evidence that HflX is involved in the resistance to transcriptional inhibitors such as rifampicin, which limits the understanding of the universal regulatory function of HflX in bacterial antibiotic resistance.

RNAP plays a central role in transcription, and consequently in gene expression, in *Brucella* (Qureshi and Duss, 2025; Woodgate and Zenkin, 2023). The RNAP holoenzyme (α2ββ’ωσ) initiates transcription under the control of multiple σ factors (RpoD, RpoH1/H2, RpoE1/E2, and RpoN) and dedicated transcription factors (Delory et al., 2006; Travis and Schumacher, 2022; Willett et al., 2016). During transcriptional elongation, NusA and NusG assist RNAP, while ribosomes engage nascent mRNA to form an expressosome-like assembly that supports efficient co-transcriptional translation (Bailey et al., 2022; Webster et al., 2020). HflX maintains translational homeostasis and cellular energetics during this process, thereby supporting the processivity of RNAP. Studies on *mycobacteria* reveal that *hflX* knock under stress results in the accumulation of stalled ribosomes and energetic stress (Ngan et al., 2021). During a stress response in numerous bacterial species, intracellular (p)ppGpp binds HflX, lowering cellular energy levels and increasing resistance to several antibiotics, including rifampicin (Bennison et al., 2021; Corrigan et al., 2016). In Proteobacteria (p)ppGpp represses energy-intensive genes, including those required for ribosome biogenesis, and redistributes σ-factor usage, thereby modulating RNAP activity (Girard et al., 2018; Gourse et al., 2018; Molodtsov et al., 2018). The direct regulatory link between HflX and RNAP expression, as well as its subsequent role in rifampicin resistance, has not been experimentally verified in any intracellular pathogen, including *Brucella*. The regulatory axis involving HflX and RNAP is likely conserved across *Brucella* species, although direct experimental confirmation is still required. These observations suggest that HflX may play a role in modulating rifampicin resistance in *Brucella*. To date, the contribution of HflX to rifampicin resistance in *Brucella* spp. remains unexplored.

Our study reveals that HflX is a key determinant of rifampicin resistance in *Brucella*. *hflX* knock not only increased bacterial susceptibility but also triggered a distinct cellular stress response, as visualized by electron microscopy, despite maintaining cell envelope integrity. At the molecular level, proteomic analysis directly linked HflX to the regulation of RNAP core subunits, suggesting its role in modulating the transcriptional machinery in response to antibiotic stress. Collectively, these findings position HflX as a promising novel target for therapeutic strategies designed to counteract rifampicin resistance in *Brucella*.

## 2 Materials and methods

### 2.1 Propagation of Brucella strains

The *Brucella abortus* strain 2308 (*B. abortus* 2308, CVCC788) preserved in our laboratory. Prior to use, the strain was activated and cultured on tryptic soy agar (TSA) plates supplemented and incubated at 37°C with 5%CO_2_ for 72 h. All investigations using live bacteria were performed in a Biosafety Level 3 (BSL-3) laboratory environment adhering to national biosafety regulations. Appropriate volumes of bacterial suspensions were used for *Brucella* genomic DNA extraction, competent-cell preparation, and serial-dilution plating on TSA plates to enumerate colony-forming units (CFU).

### 2.2 Construction of gene knock and complementation strains

The *hflX* knock strain (Δ*hflX*) was constructed by homologous recombination using the pUC19-sacB suicide plasmid. The *hflX* gene (locus corresponding to NC_007618.1) was targeted for knock. Upstream and downstream homology arms flanking the *hflX* gene were PCR-amplified from *B. abortus* 2308 wild-type (WT) genomic DNA and cloned into the linearized pUC19-sacB vector using the ClonExpress II One Step Cloning Kit (Vazyme, Nanjing, China) to construct the *hflX* knock plasmid (Table 1). The plasmid was electroporated into the recipient strain at 2.5 kV, 200 Ω, and 5.0 ms. Single-crossover transformants were selected on TSA plates containing 25 μg/mL kanamycin (Solarbio, Beijing, China) at 37°C for 3–5 days, followed by 10% (w/v) sucrose counterselection at 37°C for 5–6 days to isolate double-crossover recombinants. Δ*hflX* strain was further verified by colony PCR using the verification primers *hflX*-check-F and *hflX*-check-R, which are listed in Table 1. The successful construction of the Δ*hflX* strain was confirmed by colony PCR and DNA sequencing.

**TABLE 1:** Primers used for construction of the Δ*hflX* and CΔ*hflX* strains in *B. abortus*.

Primers	Sequences (5’–3’)	Amplicons (bp)	Source
*hflX-*Up-F1	ACCTGCAGGCATGCAAGCTTTACATGTGGGCAATAGATCGGG	700	NC_007618.1
*hflX-*Up-R1	AAAGCAACCTGTCAACGCCTGGATCTGCCTTGCAGATAAGTCACC		
*hflX*-Down-F2	CTTATCTGCAAGGCAGATCCAGGCGTTGACAGGTTGCTTT	750	NC_007618.1
*hflX*-Down-R2	ACCATGATTACGCCAAGCTTCTGGTGAACCATGATGCGCG		
*hflX*-check-F	CTCAATATCCGAAGCCTGG	2726	NC_007618.1
*hflX*-check-R	AGCATAAGGAAGTCGTGTC		
pBB-*hflX*-F	TTATCAGGCTCTGGGAGGATGCGTTCCCGCGTCCCCGG	1446	NC_007618.1
pBB-*hflX*-flag-R	ATTAAGCATTGGTAATTACTTGTCATCGTCGTCCTTGTACTACTCCCAATCGATCTCAG		

The complementation strain was constructed by cloning the full-length *hflX* coding sequence with its native promoter and a C-terminal Flag tag into the pBBR1MCS-2 plasmid. The verified recombinant plasmid was electroporated into the Δ*hflX* strain, and transformants were selected on TSA plates supplemented with kanamycin (25 μg/mL). The successful construction of the complemented strain (CΔ*hflX*) was verified by Western blotting using mouse anti-Flag monoclonal antibody.

### 2.3 Antimicrobial susceptibility testing

Antimicrobial susceptibility was assessed using the standard disk diffusion assay. Bacterial suspensions were adjusted to a concentration of 0.5 McFarland units and uniformly spread on TSA plates. Rifampicin (Solarbio, Beijing, China) disks (5 μg) were aseptically placed on the inoculated agar surface. Plates were incubated at 37°C in 5%CO_2_ for 48 h, after which inhibition zone diameters were measured in millimeters using a vernier caliper.

The minimum inhibitory concentration (MIC) was determined by broth microdilution. Rifampicin was prepared in tryptic soy broth (TSB) and two-fold serially diluted in 96-well microplates to yield a concentration range of 64–0.25 μg/mL. The test strains were cultured for 24 h and adjusted to 0.5 McFarland, then further diluted to 1 × 10^5^ CFU/mL. The 96-well plates containing the bacterial suspensions and rifampicin dilutions were incubated at 37°C for 72 h. The MIC was determined by measuring the optical density (OD) at 600 nm (OD_600_) using a microplate reader (Scientz, Zhejiang, China). The MIC was defined as the lowest drug concentration at which no visible bacterial growth was detected.

Cultures were diluted to 10^5^ CFU/mL, and 100 μL aliquots were spread onto TSA plates supplemented with rifampicin (0.5–8 μg/mL). Positive control (PC) plates contained no antibiotic; negative control (NC) plates were inoculated with sterile PBS to exclude contamination. After 5 days of incubation at 37 °C with 5 %CO_2_, colony growth was photographed and analyzed.

### 2.4 Growth analysis

Bacterial growth kinetics in the presence of rifampicin at 1/2 MIC were assessed using a growth-curve analyzer. Cultures were inoculated at a final density of 1 × 10^5^ CFU/mL into 24-well plates containing TSB supplemented with rifampicin, while an antibiotic-free well served as the control (Georges et al., 2022). The plates were then incubated at 37°C for 120 h and the OD_600_ was automatically recorded at 6 h intervals to generate growth curve.

### 2.5 Time-kill assay

Bactericidal kinetics of rifampicin at 1/2 MIC were quantified by enumeration of CFU. Bacterial suspensions at approximately 5 × 10^5^ CFU/mL were inoculated into TSB-containing rifampicin tubes and incubated at 37°C with shaking. Samples were collected at 6 h intervals from 0 to 48 h, serially diluted and spread onto TSA plates. After incubation, colonies were counted to calculate CFU/mL at each time point. Time-kill curves were generated by plotting log_10_ (CFU/mL) versus time.

### 2.6 RNA extraction, transcriptome analysis, and qRT-PCR

Total RNA was extracted using TRIzol reagent according to the manufacturer’s instructions. The concentration of total RNA was assessed using the spectrophotometer (Implen, Munich, Germany). Genomic DNA elimination was performed using gDNA Eraser from the One-Step Reverse Transcription Kit (Vazyme, Nanjing, China), followed by first-strand cDNA synthesis. qRT-PCR was performed using the primer pairs listed in Table 2 to obtain cycle threshold (Ct) values. The 16S rRNA gene was used as the internal reference gene for normalization, and its stable expression was verified across all samples. Relative transcript levels of the target genes were calculated using the 2^–ΔΔCt^ method. Transcripts of each gene were assessed in three technical replicates.

**TABLE 2:** qRT-PCR primers used in this study.

Primers	Sequences (5’–3’)	Amplicons (bp)	Source
*rpoA*-F	GTCAACACCGGCAAGGGATA	199	NC_007618.1
*rpoA*-R	CTTCACCCGTAACCGAACCA		
*rpoB*-F	AATGTGGAGCTTGACGAGCA	128	NC_007618.1
*rpoB*-R	GAAACAAGCTGCTTTGGCGA		
*rpoC*-F	TCAGAATCTCGATCGCCAGC	176	NC_007618.1
*rpoC*-R	CATACGCTTGTACTTGCCGC		
*rpoD*-F	ATGAGCTTGACCCGAACTGG	170	NC_007618.1
*rpoD*-R	CTGATTGACGATACGGCGGA		
*rpoH*-F	CCATGTGGTGGATCAAGGCT	162	NC_007618.1
*rpoH*-R	CCTGATCCGGGTTGAGATCG		

### 2.7 TEM and SEM sample preparation

The WT, Δ*hflX*, and CΔ*hflX* strains were harvested during the logarithmic growth phase after three consecutive passages in rifampicin at 1/2 MIC. Bacterial cells were collected by centrifugation and fixed in pre-cooled 2.5% (v/v) glutaraldehyde prepared in 0.1 M PBS (pH 7.4). Subsequently, samples were post-fixed in 1% osmium tetroxide and rinsed three times with deionized water. The samples were then made into ultrathin sections and viewed under TEM (Hitachi, Tokyo, Japan) with Hitachi TEM system control. For SEM (Hitachi, Tokyo, Japan) using JEOL SEM system control, strains were directly observed as a colony mass.

### 2.8 Proteome analysis

Proteomic analysis was performed on WT, Δ*hflX*, and CΔ*hflX* strains of *Brucella abortus* by Biotree (Shanghai, China). Following three passages under 1/2 MIC rifampicin exposure, cells were harvested at the logarithmic growth phase (*n* = 3 per strain). Cells were lysed in PBS by ice-bath ultrasonication at 30% power with a working/intermittent cycle of 3 s on/5 s off, for a total duration of 20 min. These ultrasonic parameters were optimized through preliminary experiments to ensure efficient cell lysis and complete protein release, while minimizing protein denaturation and degradation—key considerations for maintaining the integrity and activity of proteins for subsequent LC-MS/MS analysis. The cell lysates were clarified by centrifugation (12,000 rpm, 4°C, 10 min). Protein concentration was determined using the bicinchoninic acid (BCA) assay. For each sample, 30 μg of protein was processed using the SP3 workflow, involving reduction, alkylation, bead-based capture, washing, and tryptic digestion. The resulting peptides were desalted, vacuum-dried, and reconstituted for Liquid Chromatography-Mass Spectrometry (LC-MS)/Mass Spectrometry (MS) analysis.

Peptide separation was performed on a nano-UPLC Vanquish Neo system coupled with an EASY-Spray C18 column (150 μm × 15 cm), using a gradient of buffer A (0.1% formic acid in water) and buffer B (80% acetonitrile/0.1% formic acid). MS data were acquired on an Astral mass spectrometer in Data-Independent Acquisition (DIA) and positive-ion mode (Full MS m/z 380–980, 240 k resolution; isolation window 2 m/z; NCE 25%; MS/MS m/z 150–2000).

Raw data were processed using Spectronaut (v19.0.240604.62635) with its built-in Pulsar engine against a *Brucella abortus* UniProt FASTA database. Search parameters included trypsin as the protease (max. 2 missed cleavages), carbamidomethylation as a fixed modification, and oxidation and acetylation (Protein N-term) as variable modifications. Precursor and fragment mass tolerances were both set to 20 ppm. The false discovery rate (FDR) was controlled at 1% for both peptide-spectrum matches (PSMs) and peptides. All other parameters were kept as default.

### 2.9 Statistical analysis

Statistical analyses were performed using GraphPad Prism 8 software. Data are presented as mean ± standard deviation (SD) from at least three independent biological experiments. Differences among groups were evaluated using one-way analysis of variance (ANOVA) by Tukey’s multiple-comparison test, with *p* < 0.05 considered statistically significant.

## 3 Results

### 3.1 The gene *hflX* knock in *B. abortus*

**FIGURE 1:**
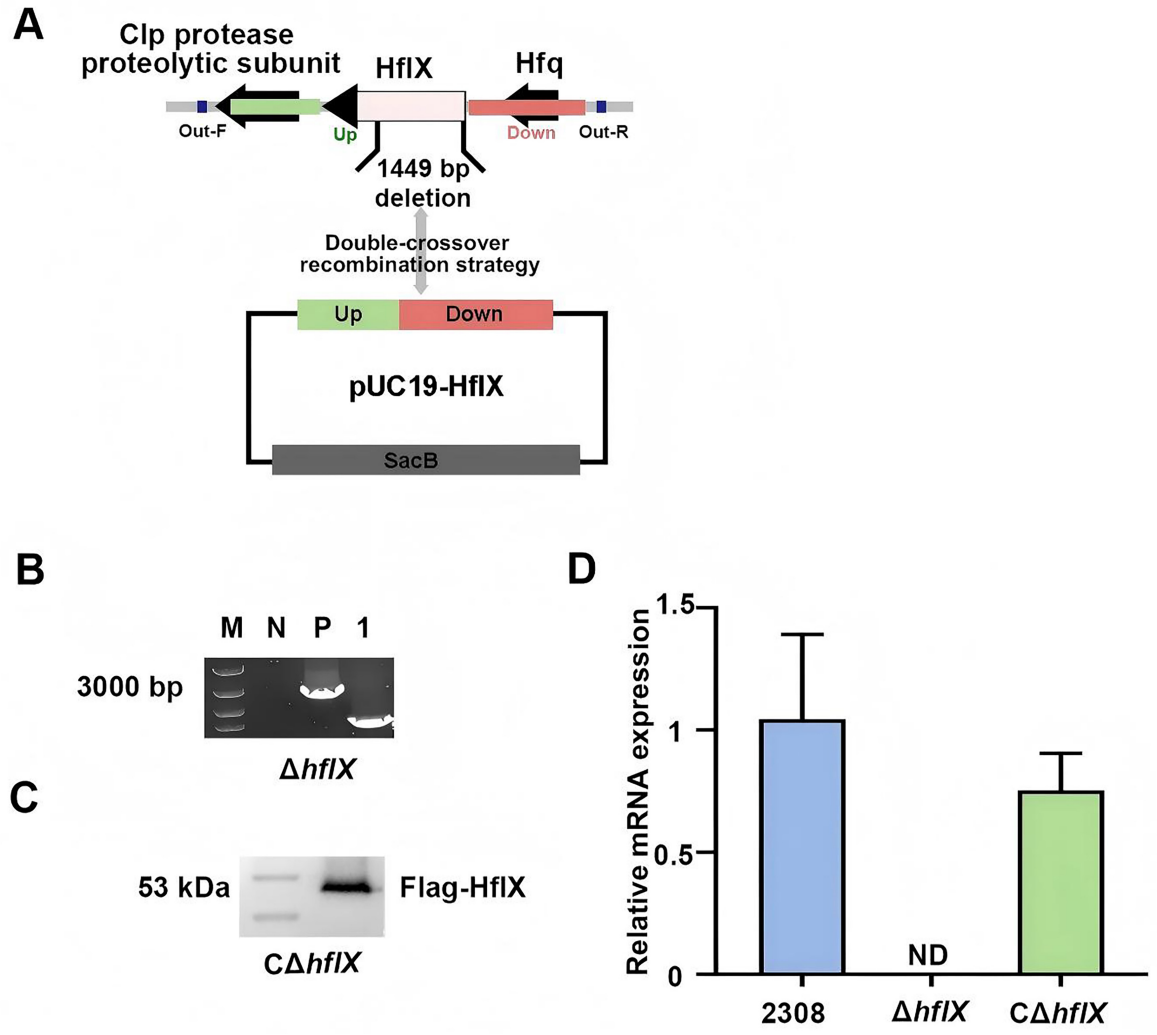
Screening and identification of *hflX* as a rifampicin-resistance gene in *B. abortus*. **(A)** Construction of the Δ*hflX* strain of *B. abortus*. **(B)** PCR verification of the Δ*hflX* strain: lane N: negative control; lane P, WT strain; lane 1, Δ*hflX* strain. **(C)** Western blot verification of the HflX complementation strain. **(D)** Relative expression levels of *hflX* in different strains, with the WT strain used as the reference. ND, Not detected.

To assess the role of HflX in rifampicin susceptibility, we constructed an *hflX*-knock mutant (Δ*hflX*) of *B. abortus* 2308 through homologous recombination (Figure 1A). PCR analysis verified the successful construction of the Δ*hflX* strain, as the *hflX* signal was not detected in the mutant strain compared with the WT strain (Figure 1B). Subsequently, we constructed a CΔ*hflX* by reintroducing a Flag-tagged *hflX* gene, which restored protein expression, as verified by Western blotting (Figure 1C). RT-qPCR analysis confirmed that transcription of *hflX* was significantly downregulated in the Δ*hflX* strain compared to the WT strain, and expression of HflX was restored in the complement strain (Figure 1D). These results confirm the successful construction of both the knock and complemented strains, providing a reliable genetic basis for subsequent investigations.

### 3.2 HflX deficiency enhances *B. abortus* susceptibility to rifampicin

**FIGURE 2:**
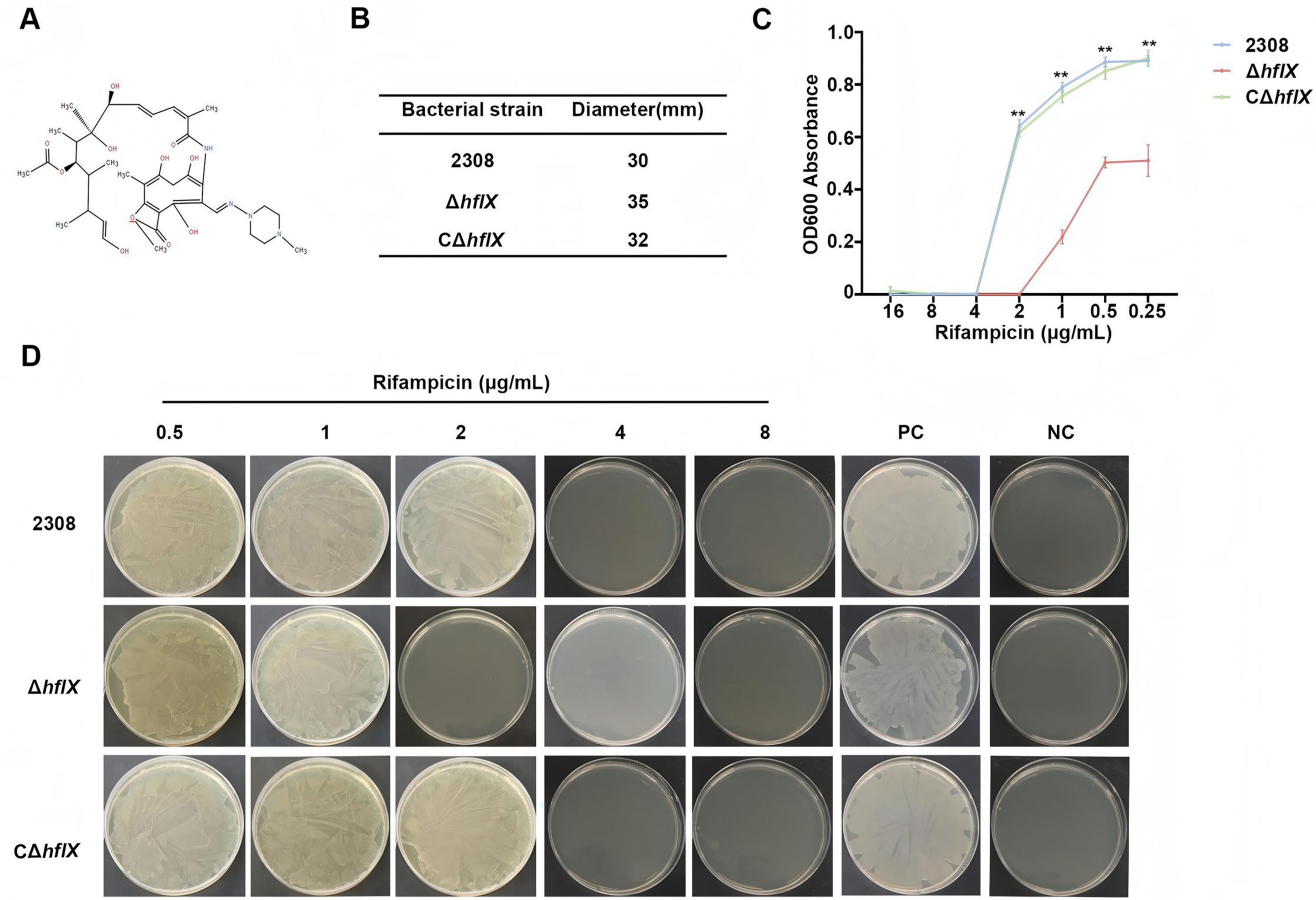
Rifampicin structure and antimicrobial susceptibility profiles of *Brucella* strains. **(A)** Chemical structure of rifampicin. **(B)** Results of the standard disk diffusion assay for the three strains. **(C)** MIC values of rifampicin for the different strains, (WT/CΔ*hflX*: 4 μg/mL, Δ*hflX*: 2 μg/mL). **(D)** Growth inhibition on TSA plates supplemented with increasing concentrations of rifampicin. PC, positive control. NC, negative control. Significance levels: **p < 0.01.

To further evaluate the role of HflX in rifampicin sensitivity (chemical structure shown in Figure 2A), we conducted Kirby-Bauer disk diffusion assays. Compared with the WT strain (30 mm) and the CΔ*hflX* strain (32 mm), the Δ*hflX* strain exhibited a significantly larger zone of inhibition (35 mm) (Figure 2B), indicating increased sensitivity to rifampicin following *hflX* knock. To more precisely quantify the observed difference, we sought to determine the MIC of rifampicin of each strain using a broth microdilution assay. Bacterial strains were cultured in 96-well plates containing serial dilutions of rifampicin, and growth was monitored by measuring OD_600_. The MIC values of the WT and CΔ*hflX* strains were 4 μg/mL, whereas that of the Δ*hflX* strain was markedly decreased to 2 μg/mL (Figure 2C), demonstrating an increased susceptibility to rifampicin with *hflX* knock. These findings were further validated by assessing growth of the bacterial strains on TSA plates supplemented with increasing concentrations of rifampicin. We found that the Δ*hflX* strain was completely inhibited at lower concentrations of rifampicin as compared to the WT and CΔ*hflX* strains (Figure 2D). These results confirm that *hflX* knock increases rifampicin susceptibility and that resistance is restored with complementation.

### 3.3 HflX alters rifampicin resistance in *Brucella* without affecting bacterial proliferation

**FIGURE 3:**
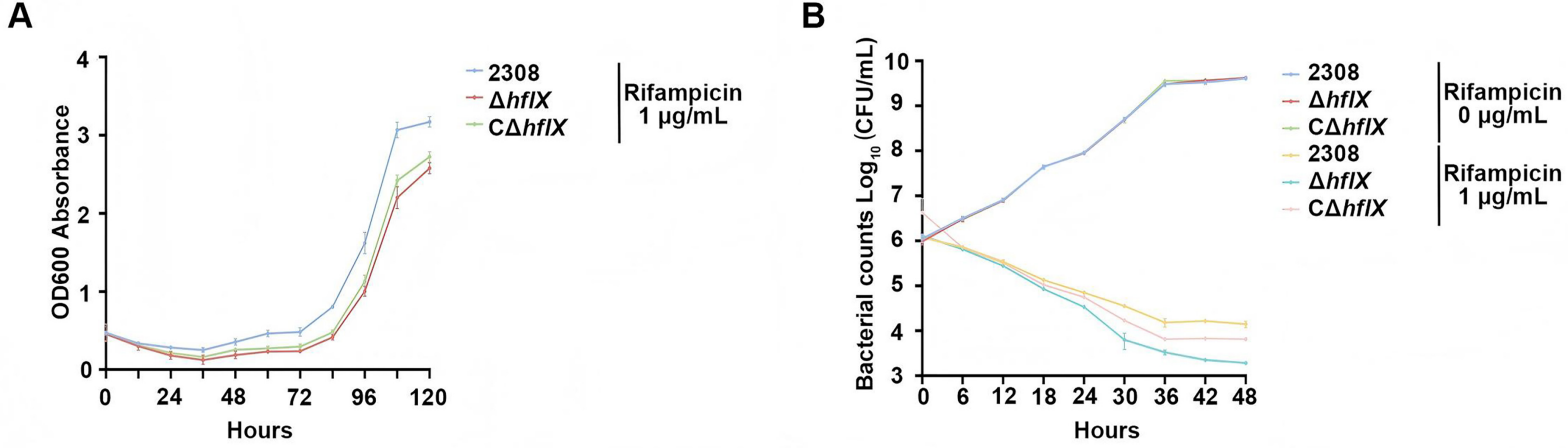
Growth curve and CFU assays assessing the impact of *hflX* deletion on bacterial growth and survival. **(A)** Growth curve of the WT, Δ*hflX*, and CΔ*hflX* strains under 1/2 MIC rifampicin exposure. **(B)** CFU counts of the three strains under 1/2 MIC rifampicin, measured at 6 h intervals.

To further investigate the role of HflX in rifampicin exposure response, we performed a time-course assay to monitor the growth and survival of the WT, Δ*hflX*, and CΔ*hflX* strains at 1/2 MIC rifampicin (1 μg/mL) over a 120 h period. The growth curve (Figure 3A) showed that all three strains exhibited a prolonged lag phase when grown in medium containing 1/2 MIC rifampicin (1 μg/mL). This result indicated substantial early growth inhibition by the antibiotic on all strains, particularly within the first 48 h. However, the most pronounced inhibitory effect was observed in the Δ*hflX* strain. At later time points, the WT and CΔ*hflX* strains recovered more rapidly, reaching OD_600_ values of approximately 3.3 and 3.0 at 120 h, respectively. In contrast, the Δ*hflX* strain consistently showed lower OD_600_ than the other two strains across all time points, indicating impaired growth recovery under rifampicin exposure (1 μg/mL) and increased drug susceptibility.

To further evaluate the bactericidal kinetics under antibiotic exposure, CFU assays were performed for the three strains in the absence or presence of rifampicin (1 μg/mL) at 6 h intervals over a 48 h period. In antibiotic-free medium, all strains exhibited exponential growth, indicating that Δ*hflX* does not impair bacterial proliferation under normal growth conditions. In contrast, rifampicin exposure (1 μg/mL) resulted in decreased CFU counts over time in all three strains, though to varying degrees. Uniquely, the Δ*hflX* strain exhibited a more rapid decline in viable counts than the WT and CΔ*hflX* strains. While the Δ*hflX* strain showed CFU counts of 10^3^CFU/mL at 48 h, the WT and CΔ*hflX* strains exhibited greater survival under the same conditions, with CFU counts maintained at 10^5^CFU/mL at 48 h (Figure 3B). These data support the hypothesis that the expression of HflX is essential for rifampicin resistance in *Brucella*.

### 3.4 *hflX* knock heightens cellular stress without compromising envelope integrity

**FIGURE 4:**
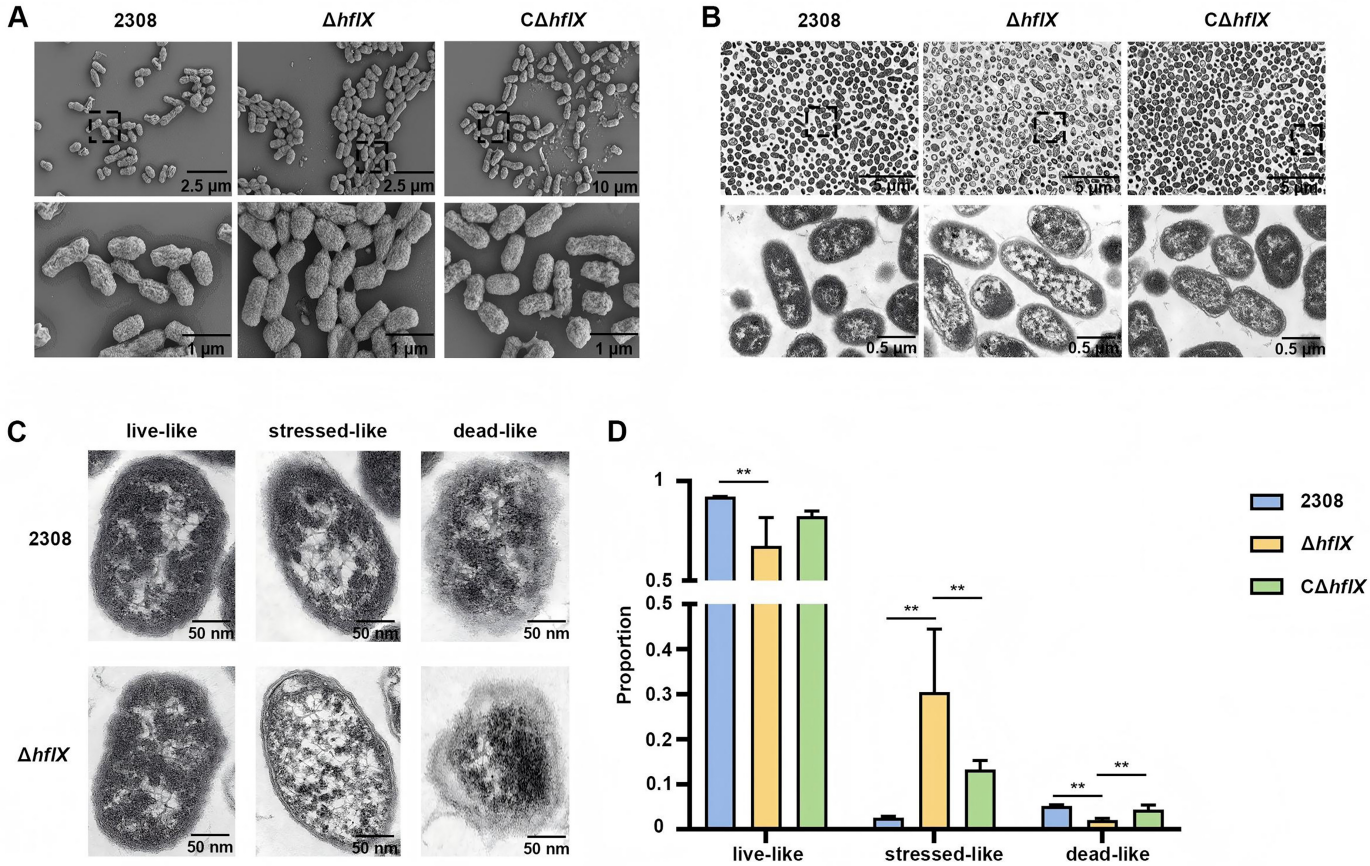
SEM and TEM analyses under 1/2 MIC rifampicin assessing the impact of *hflX* deletion on bacterial stress phenotypes. **(A)** Representative SEM images assessing bacterial membrane integrity, showing that the *hflX* deletion does not compromise membrane integrity. **(B)** Representative TEM images revealing an increased stress-associated phenotype in the Δ*hflX* strain compared with the WT strain. **(C,D)** Quantification of the proportions of the WT, Δ*hflX* and CΔ*hflX* strains in three distinct morphological states based on TEM analysis. Significance levels: **p < 0.01.

To explore the mechanism underlying the increased rifampicin susceptibility observed upon *hflX* knock, we compared the ultrastructural features of the WT and Δ*hflX* strains after three passages in 1/2 MIC rifampicin (1 μg/mL) using scanning SEM and TEM, followed by quantitative analysis. SEM analysis revealed that both the WT and Δ*hflX* strains largely retained intact rod-shaped morphology with smooth, continuous surfaces and no obvious membrane rupture (Figure 4A). The outer membrane integrity of the Δ*hflX* strain was comparable to that of the WT strain, indicating that *hflX* knock does not disrupt gross envelope integrity. In contrast, TEM analysis revealed pronounced intracellular differences between the two strains. As shown in Figure 4B, the WT cells predominantly displayed intact ultrastructure with a homogeneous, electron-dense cytoplasm. Conversely, a substantial proportion of Δ*hflX* strain exhibited stress-associated morphological features in the presence of rifampicin.

To quantitatively assess these phenotypes, bacteria were classified into three categories based on envelope integrity and cytoplasmic electron density according to criteria previously established for *Brucella* (Georges et al., 2022): live-like, characterized by an intact envelope, uniform periplasmic space, and homogeneous cytoplasm with evenly distributed ribosome-like particles; stressed-like, characterized by preserved envelope continuity but with periplasmic undulations, heterogeneous cytoplasmic density, particle aggregation, and focal vacuole-like structures; dead-like, characterized by disrupted envelope integrity, collapsed cell outlines, and diffuse cytoplasmic contents indicative of leakage. Under identical conditions, visual inspection revealed a markedly higher proportion of stress-associated morphotypes in the Δ*hflX* strain compared with the WT strain (Figure 4C). Quantitative analysis confirmed a significant increase in stressed-like cells and a corresponding decrease in live-like cells in the Δ*hflX* group (Figure 4D). Although the proportion of dead-like cells remained low in both groups, a modest increase was observed in the Δ*hflX* strain. Together, these results indicate that under 1/2 MIC rifampicin, *hflX* knock exacerbates intracellular stress without causing overt envelope disruption. Rather than inducing direct cell lysis, *hflX* knock drives bacteria toward severe structural disorganization, suggesting that HflX buffers rifampicin-induced stress and supports cellular homeostasis.

### 3.5 *hflX* knock reduces the abundance of RNAP-associated proteins

**FIGURE 5:**
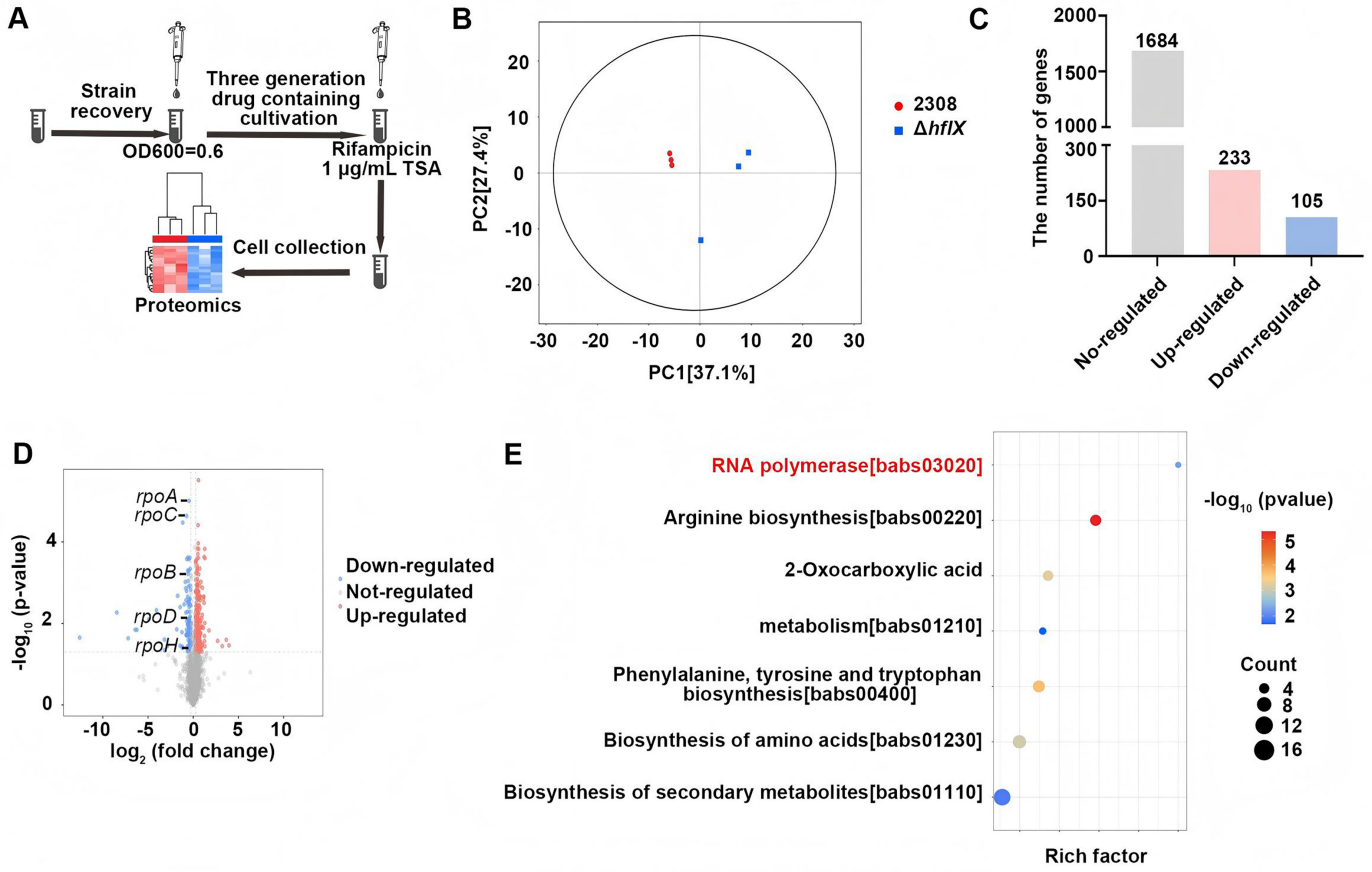
RNAP and metabolic impact of *hflX* deletion in *B. abortus* revealed by proteomics analysis. **(A)** Workflow for proteomic sample preparation. Strains were cultured for three generations under 1/2 MIC rifampicin exposure and harvested during the logarithmic phase for DIA-MS proteomic profiling. **(B)** PCA score plot of proteomic profiles showing separation between the WT and Δ*hflX*; PC1 and PC2 account for 37.1 and 27.4% of total variance, respectively. **(C)** Differentially expressed protein counts for Δ*hflX* compared to the WT (233 upregulated and 105 downregulated proteins). **(D)** Volcano plot showing differentially expressed proteins in the Δhflx strain compared with the WT strain. **(E)** KEGG pathway enrichment analysis of significantly downregulated proteins.

To further investigate molecular changes associated with *hflX* knock during rifampicin exposure, we performed label-free quantitative proteomic analysis. The WT and Δ*hflX* strains were cultured for three generations in the presence of 1/2 MIC rifampicin (1 μg/mL) and harvested during the logarithmic growth phase, with at least three biological replicates per group. DIA-based proteomic analysis quantified protein abundance and identified differentially expressed proteins (DEPs) (Figure 5A). Bioinformatic analyses were then conducted to evaluate enriched pathways and co-expression networks, aiming to elucidate the regulatory role of HflX in *Brucella*.

Principal component (PC) analysis demonstrated clear separation between the WT and Δ*hflX* samples, accounting for 64.5% of total variance (PC1: 37.1%; PC2: 27.4%). Replicates within each group clustered closely, although one Δ*hflX* replicate displayed moderate deviation, suggesting limited biological or technical variability. Importantly, the WT and Δ*hflX* clusters were clearly separated along PC1, indicating that *hflX* knock was the primary driver of proteomic variation (Figure 5B). Proteomic profiling revealed substantial differences between the WT and Δ*hflX* strains. Specifically, 233 proteins were significantly upregulated, whereas 105 proteins were significantly downregulated in the Δ*hflX* strain (Figure 5C). Volcano plot analysis revealed that downregulated proteins exhibited larger fold changes and higher statistical confidence than upregulated proteins, indicating a pronounced inhibitory signature in the mutant strain (Figure 5D). Kyoto Encyclopedia of Genes and Genomes (KEGG) pathway enrichment analysis further showed that downregulated proteins were significantly enriched in several pathways, with particularly strong enrichment observed for proteins associated with the RNAP pathway in the Δ*hflX* strain compared with the WT strain (Figure 5E). Additionally, metabolic pathways (including arginine biosynthesis, 2-oxoglutarate metabolism, and aromatic amino acid biosynthesis) were significantly affected. The coordinated downregulation of RNAP-related and metabolic pathways under rifampicin exposure suggests impaired transcriptional and metabolic adaptation, providing a mechanistic explanation for the increased rifampicin susceptibility observed in the Δ*hflX* strain.

### 3.6 *hflX* knock reduces transcript levels of RNAP-associated genes

**FIGURE 6:**
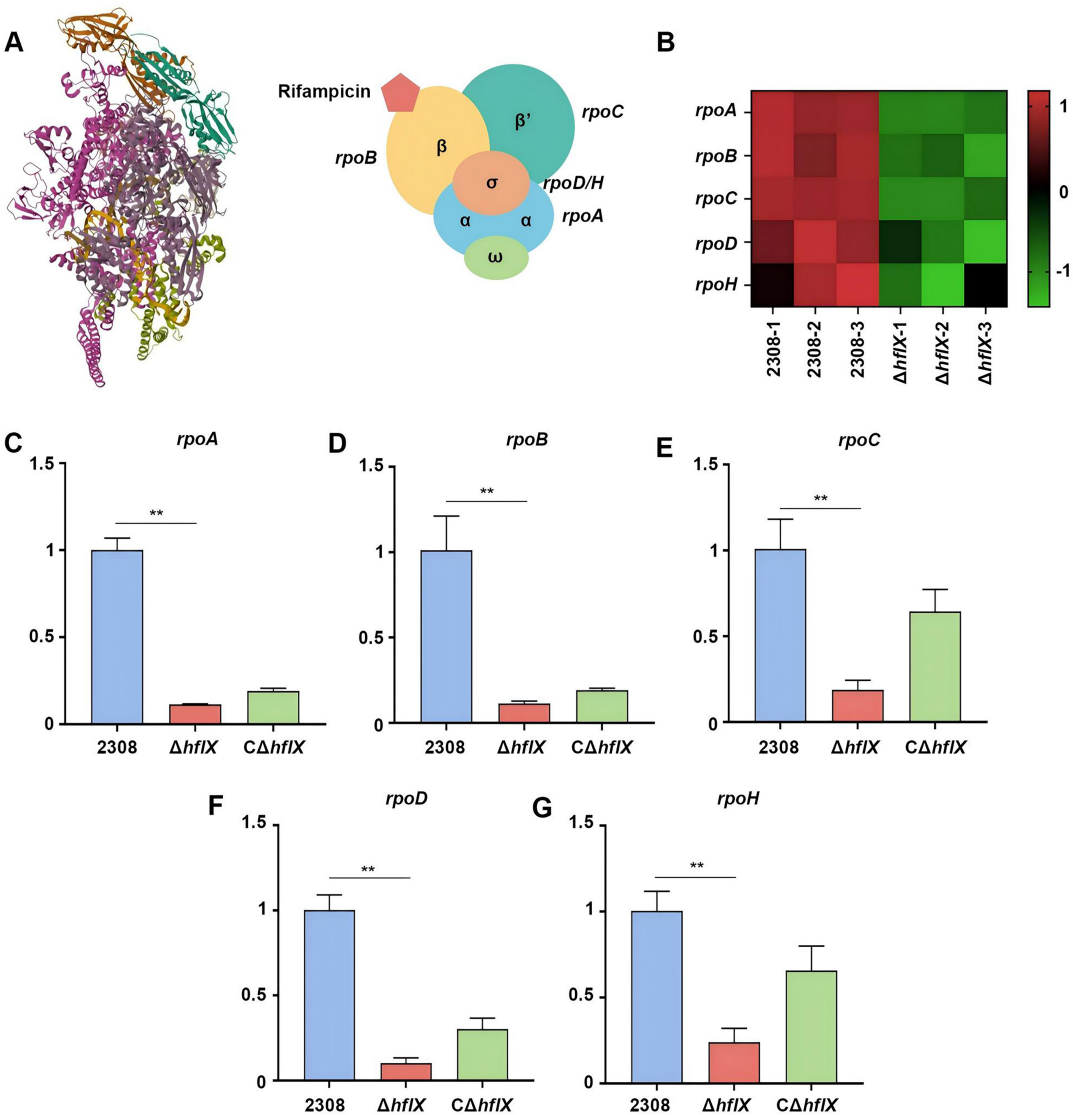
RT-qPCR validation of altered transcript levels of RNA polymerase-associated genes following *hflX* deletion. **(A)** Molecular interactions between rifampicin and *Brucella* RNAP in *Brucella*. **(B)** Heatmap showing the relative abundance of RNAP-related proteins in the WT and Δ*hflX* strains. **(C–G)** Relative transcript levels of RNAP-associated genes in different strains. Significance levels: **p < 0.01.

As illustrated in Figure 6A, the *Brucella* RNAP core enzyme consists of α2ββ′ω subunits and associates with σ factors to form the holoenzyme. Rifampicin binds to a conserved site within RpoB, thereby blocking early transcript elongation and inducing transcriptional stress. Under 1/2 MIC rifampicin conditions, DIA-MS-based proteomic heatmap analysis revealed that the abundances of *rpoA*, *rpoB*, *rpoC*, *rpoD*, and *rpoH* were consistently reduced in the Δ*hflX* strain compared with the WT strain (Figure 6B), indicating that *hflX* knock leads to downregulation of RNAP core subunits and σ-factor-associated proteins. These observations are consistent with the KEGG enrichment results for the RNAP pathway.

To determine whether these proteomic changes were reflected at the transcriptional level, we performed RT-qPCR analysis to quantify transcript levels of RNAP subunit and σ factor genes (Figures 6C–G). The results confirmed that transcripts encoding RNAP core subunits *rpoA* (α), *rpoB* (β), *rpoC* (β′), as well as σ factors *rpoD* (σ70) and *rpoH* (σ32), were significantly reduced in the Δ*hflX* strain compared with the WT strain. The complemented strain exhibited partial restoration of these transcript levels. Collectively, these data demonstrate that *hflX* knock suppresses expression of RNAP-associated genes. These findings support a model in which loss of HflX compromises RNAP-related functions and disrupts transcriptional homeostasis under antibiotic exposure, thereby enhancing rifampicin susceptibility.

## 4 Discussion

Brucellosis is a worldwide zoonotic disease caused by *Brucella* spp. (O’Callaghan, 2020). Rifampicin is a cornerstone of first-line combination therapy for brucellosis and exerts its bacteriostatic activity by binding to RpoB, thereby blocking transcriptional initiation and early elongation (Campbell et al., 2001; Sudzinová et al., 2023). However, prolonged treatment courses, recurrent infection, and chronic disease all serve as sources of rifampicin resistance in *Brucella* (Bosilkovski et al., 2021; Maduranga et al., 2024; Salehi et al., 2023). However, comprehensive mechanistic studies investigating the molecular drivers of resistance remain limited. Here, we identify HflX as a novel factor contributing to rifampicin resistance in *Brucella* and show that it functions by modulating RNAP-associated pathways, a novel mechanism that expands our understanding of *Brucella* rifampicin resistance from the traditional mutation and membrane transport-related pathways to the ribosomal quality control and transcriptional regulation-related pathways.

HflX is a highly conserved ribosome-associated GTPase that plays a crucial role in ribosome splitting and reactivation (Fischer et al., 2012; Zhang et al., 2015). In diverse bacterial species, HflX facilitates recovery of translational processes under diverse stresses, including heat, oxidative stress, and acidic conditions, and helps reset translation initiation to maintain continuous protein synthesis (Bhattacharjee et al., 2023; Sengupta et al., 2018). Recent studies have identified HflX as a ribosome rescue factor in bacteria such as mycobacteria (including non-tuberculous species) and Listeria. HflX dissociates ribosomal complexes stalled by stress or antibiotics, thereby maintaining protein synthesis rates and ribosome homeostasis (Ngan et al., 2021; Seely et al., 2024). This activity contributes to the resistance of bacteria to various protein synthesis inhibitors, including macrolides, lincosamides, chloramphenicol, and aminoglycosides (Koller et al., 2022). Structural and functional studies in mycobacteria reveal that under antibiotic stress, the N-terminal extension (NTE) of HflX induces persistent structural disorder in multiple 23S rRNA helices. This NTE-mediated remodeling facilitates 70S ribosome splitting, generating a pool of inactive 50S subunits (Zhang et al., 2015). Through this mechanism, ribosome splitting functionally buffers the effects of translation inhibitors, thereby maintaining intracellular protein synthesis and enhancing resistance to antibiotics. However, a role for HflX in rifampicin resistance has not been reported. Our study provides the first evidence that HflX modulates RNAP-related processes to influence rifampicin resistance in *Brucella*. We constructed *hflX* knock and complemented strains and found that knock of HflX significantly altered the resistance phenotype. Disk diffusion assays and MIC testing showed larger zones of inhibition and reduced MICs with *hflX* knock. Growth curve analyses indicated that *hflX* knock increased drug sensitivity without affecting bacterial proliferation, whereas time-kill assays demonstrated a significant, time-dependent decline in cell viability (CFU/mL) with rifampicin exposure. Scanning and transmission electron microscopy, combined with quantitative analyses, found that antibiotic stress did not result in any significant defects in cell envelope integrity; rather, the *hflX* knock exhibited a heightened stress response, as measured by live-like, stressed-like, and dead-like cell morphotypes. A plausible mechanism to explain these results is that antibiotic-induced ribosome stalling increases translational stress and proteostatic burden, thereby activating stress-response pathways and enhancing susceptibility to antibiotics. Consistent with this interpretation, proteomic profiling revealed reduced abundance of RNAP-associated proteins in the Δ*hflX* strain, and RT-qPCR analysis confirmed decreased transcript levels of RNAP core subunits *rpoA* (α), *rpoB* (β), *rpoC* (β’), and sigma factors *rpoD* (σ^70^) and *rpoH* (σ^32^). Together, these findings indicate that the *hflX* knock increases rifampicin susceptibility by enhancing the bacterial stress response and perturbing RNAP-related pathways, without impairing baseline growth rate.

Antimicrobial resistance poses a major challenge to the effective treatment of bacterial infections by substantially diminishing antibiotic efficacy. Brucellosis remains a persistent global health concern, where emerging resistant strains escalate both medical and socioeconomic burdens and intensify public health risks (Abbas et al., 2024). In this study, we comprehensively investigate rifampicin resistance mechanisms in *Brucella* spp., due to its clinical importance in brucellosis therapy. Current evidence indicates that the predominant mechanism of rifampicin resistance involves mutations within the rifampicin-resistance-determining region (RRDR) of the RNAP β subunit (*rpoB*) (Patel et al., 2023). Other mechanisms of resistance include efflux pump activation, altered membrane permeability, and adaptive metabolic and stress response, which collectively enhance rifampicin-resistant phenotype (Meng et al., 2025; Rouvier et al., 2025; Yang et al., 2020). In this work, we identify HflX as a critical determinant of rifampicin resistance, providing new insights into the underlying mechanisms. Our findings indicate that *hflX* knock increases rifampicin susceptibility in *Brucella* through two interconnected mechanisms: first, under rifampicin-induced stress, *hflX* knock exacerbates intracellular stress response, likely due to accumulation of stalled ribosomes, disrupted translation, and impaired protein homeostasis; second, *hflX* knock perturbs RNAP function by reducing the abundance of RNAP-associated proteins and transcripts of RNAP-related genes. Collectively, these pathways converge to reduce bacterial tolerance to rifampicin.

Based on our findings in *Brucella* and evidence from other bacterial systems, we propose several directions for future investigation of HflX-mediated rifampicin resistance. HflX may facilitate dissociation of stalled ribosomes, thereby maintaining translation and stabilizing the NusG-dependent expressome (RNAP-ribosome complex) (O’Reilly et al., 2020; Washburn et al., 2020). Such stabilization could enhance RNAP processivity and partially overcome rifampicin-induced inhibition of early transcriptional elongation (Kohler et al., 2017; Proshkin et al., 2010). In addition, the alarmone (p)ppGpp can bind HflX and inhibit its GTPase activity during stress-induced stringent responses, thereby limiting HflX-mediated ribosome splitting and 50S subunit biogenesis (Bennison et al., 2021; Corrigan et al., 2016). This process conserves cellular energy and downshifts global translation and proteostasis programs, potentially contributing to rifampicin tolerance (Salzer and Wolz, 2023). Although such mechanisms have been described in *Bacillus species* (e.g., *B. anthracis*) and Staphylococcus aureus, they remain to be experimentally validated in *Brucella*. Future studies should also explore whether HflX modulates susceptibility to other RNAP-targeting antibiotics.

## 5 Conclusion

In conclusion, *hflX* knock in *Brucella* results in a heightened cellular stress response and impaired RNAP-associated functions, thereby increasing sensitivity to rifampicin. This study is the first to systematically elucidate the molecular mechanism of HflX-mediated rifampicin resistance in *Brucella*, and the identified HflX-RNAP regulatory axis provides a novel theoretical basis for understanding the adaptive resistance of intracellular pathogens to transcriptional inhibitors. These findings provide a strong rationale for the development of novel HflX-targeted therapeutics to combat rifampicin-resistant brucellosis, and HflX as a novel drug target is expected to solve the clinical problem of poor curative effect of traditional antibiotics on rifampicin-resistant *Brucella*, providing a new direction for the development of anti-brucellosis drugs.

## Data Availability

The mass spectrometry proteomics data have been deposited to the ProteomeXchange Consortium (https://proteomecentral.proteomexchange.org) via the iProX partner repository with the dataset identifier PXD075846.
